# Anti-inflammatory role of SGLT2 inhibitors as part of their anti-atherosclerotic activity: Data from basic science and clinical trials

**DOI:** 10.3389/fcvm.2022.1008922

**Published:** 2022-09-06

**Authors:** Lucia Scisciola, Vittoria Cataldo, Fatemeh Taktaz, Rosaria Anna Fontanella, Ada Pesapane, Puja Ghosh, Martina Franzese, Armando Puocci, Antonella De Angelis, Liberata Sportiello, Raffaele Marfella, Michelangela Barbieri

**Affiliations:** ^1^Department of Advanced Medical and Surgical Sciences, University of Campania “Luigi Vanvitelli”, Naples, Italy; ^2^Department of Experimental Medicine, Section of Pharmacology “L. Donatelli”, University of Campania “Luigi Vanvitelli”, Naples, Italy; ^3^Mediterranea Cardiocentro, Napoli, Italy

**Keywords:** SGLT2 inhibitors (SGLT2i), SGLT2, atherosclerosis, atherosclerosis cardiovascular diseases, inflammation

## Abstract

Atherosclerosis is a progressive inflammatory disease leading to mortality and morbidity in the civilized world. Atherosclerosis manifests as an accumulation of plaques in the intimal layer of the arterial wall that, by its subsequent erosion or rupture, triggers cardiovascular diseases. Diabetes mellitus is a well-known risk factor for atherosclerosis. Indeed, Type 2 diabetes mellitus patients have an increased risk of atherosclerosis and its associated-cardiovascular complications than non-diabetic patients. Sodium-glucose co-transport 2 inhibitors (SGLT2i), a novel anti-diabetic drugs, have a surprising advantage in cardiovascular effects, such as reducing cardiovascular death in a patient with or without diabetes. Numerous studies have shown that atherosclerosis is due to a significant inflammatory burden and that SGLT2i may play a role in inflammation. In fact, several experiment results have demonstrated that SGLT2i, with suppression of inflammatory mechanism, slows the progression of atherosclerosis. Therefore, SGLT2i may have a double benefit in terms of glycemic control and control of the atherosclerotic process at a myocardial and vascular level. This review elaborates on the anti-inflammatory effects of sodium-glucose co-transporter 2 inhibitors on atherosclerosis.

## Introduction

Atherosclerosis is a widespread chronic inflammatory disorder of large- and medium-caliber artery walls with a complex biochemical and cellular etiology (1). It is characterized by the accumulation of immune-system and endothelial cells, lipid particles, and extracellular matrix components in the sub-endothelial layer (2). In the final stage, atherosclerosis exhibits the development of plaques at the intimal layer of the arterial wall, triggering cardiovascular diseases (CVDs) caused by subsequent erosion or rupture of the atherosclerotic plaques (3).

Therefore, to date, it still represents the major potential risk factor of most CVDs, including myocardial infarction (MI), heart failure (HF), stroke, and peripheral arterial disease, causing disability and mortality worldwide (4).

Cardiovascular diseases are the leading cause of death globally. Over 17.9 million people died yearly from CVDs in 2019, representing 32% of all deaths worldwide. 85% of these deaths were due to heart attack and stroke (5). Globally, over three-quarters of CVDs deaths occur in low- and middle-income countries because individuals with CVDs have limited healthcare access, delaying CVDs detection and increasing mortality from CVDs. CVDs lead to 18% of disability-adjusted life years lost in high-income countries and 10% in low-income and middle-income countries, placing a heavy burden on the economies of developing countries (5, 6). CVDs account for 62% of European healthcare costs of 169 billion euros annually (6), and most countries cannot maintain such costs. Adopting a healthy lifestyle such as reducing tobacco and alcohol, avoiding an unhealthy diet, and performing physical activity should markedly reduce the atherosclerosis burden and its complications. However, it is essential to detect cardiovascular diseases as soon as possible to begin managing them with counseling and medicines (7).

Atherosclerosis results from predisposing genetic factors and exposure to oxidative and inflammatory damage mediators. In addition, modifiable and non-modifiable risk factors, which include age, sex, cigarette smoking, unhealthy diet, physical inactivity, dyslipidemia, diabetes mellitus, hypertension, and obesity, contribute to its beginning and development (8). Diabetes mellitus is a well-known risk factor for atherosclerosis. Subjects affected by type 2 diabetes mellitus (T2DM) have an increased risk of atherosclerosis and its associated-cardiovascular complications than non-diabetic patients (9). To date, a new class of anti-hyperglycemic drugs, sodium-glucose co-transport 2 inhibitors (SGLT2i), represent a therapeutic novelty essential for their pleiotropic effects. In addition to glycemic control, SGLT2i is known for the cardio and nephron-protective role and, more recently, for the anti-inflammatory effect. Several experimental results have shown that SGLT2i, with suppression of inflammation, slows the progression of atherosclerosis (10).

This review reports experimental and clinical evidence to clarify the anti-inflammatory mechanisms underlying the anti-atherosclerotic effect of SGLT2i.

## Methods

A literature search was conducted by searching updated and relevant publications on SGLT2i and atherosclerosis in databases, including PubMed and Google Scholar. During this research, keywords such as “SGLT2 inhibitor, empagliflozin, dapagliflozin, canagliflozin, ertugliflozin, ipragliflozin, luseogliflozin, sotagliflozin, atherosclerosis, atherosclerosis cardiovascular diseases, inflammation, coronary heart disease, stroke, angina pectoris, myocardial infarction and peripheral artery disease, major adverse cardiac events (MACE), cardiovascular (CV) mortality” were used. *In vitro* and animal studies, clinical trials, reviews, meta-analyses, and guidelines were reviewed. We evaluated the anti-inflammatory effects of SGLT2i from experimental evidence (*in vitro* and animal models) and clinical trials. For the clinical trials, we considered the cardiovascular outcomes, including myocardial infarction, stroke, peripheral artery disease, cardiovascular death, or hospitalization for heart failure. Articles not in the English language or meeting abstracts were excluded.

## Atherosclerosis: From inflammation to complications

Atherosclerosis disease can be schematized in three stages, starting from the endothelium activation/dysfunction and resulting in plaque formation (1).

In the initial stages of the lesion, endothelial dysfunction occurs under harmful stimuli such as hypertension, dyslipidemia, and disturbed shear stress resulting in a chronic inflammatory state. Parallel changes in endothelial permeability promote oxidation of low-density lipoproteins (LDL), followed by infiltration of monocytes in the intimal layer (11). Oxidized-LDL (ox-LDL) promotes damage-associated molecular patterns (DAMPs) secretion that initialize an innate immune response by Toll-like receptors (TLR). Ox-LDL accumulation induces the expression of adhesion molecules as vascular cell adhesion molecule 1 (VCAM-1) by endothelial cells, which recall other monocytes and leukocytes (12). In turn, monocytes transform themselves into activated macrophages altering the ratio between M1 and M2 macrophages. Specifically, based on the macrophage activation process, M1 macrophages are activated in response to TLR ligands, interferons, lipopolysaccharides (LPS), and lipoproteins and express the main pro-inflammatory molecules IL (interleukins)-1β, IL-6, and TNF-α (tumor necrosis factor-α) (13). Therefore, M1 macrophages play significant roles in maintaining chronic inflammation, forming foam cells, and plaque initiation and progression (14).

**Figure 1:**
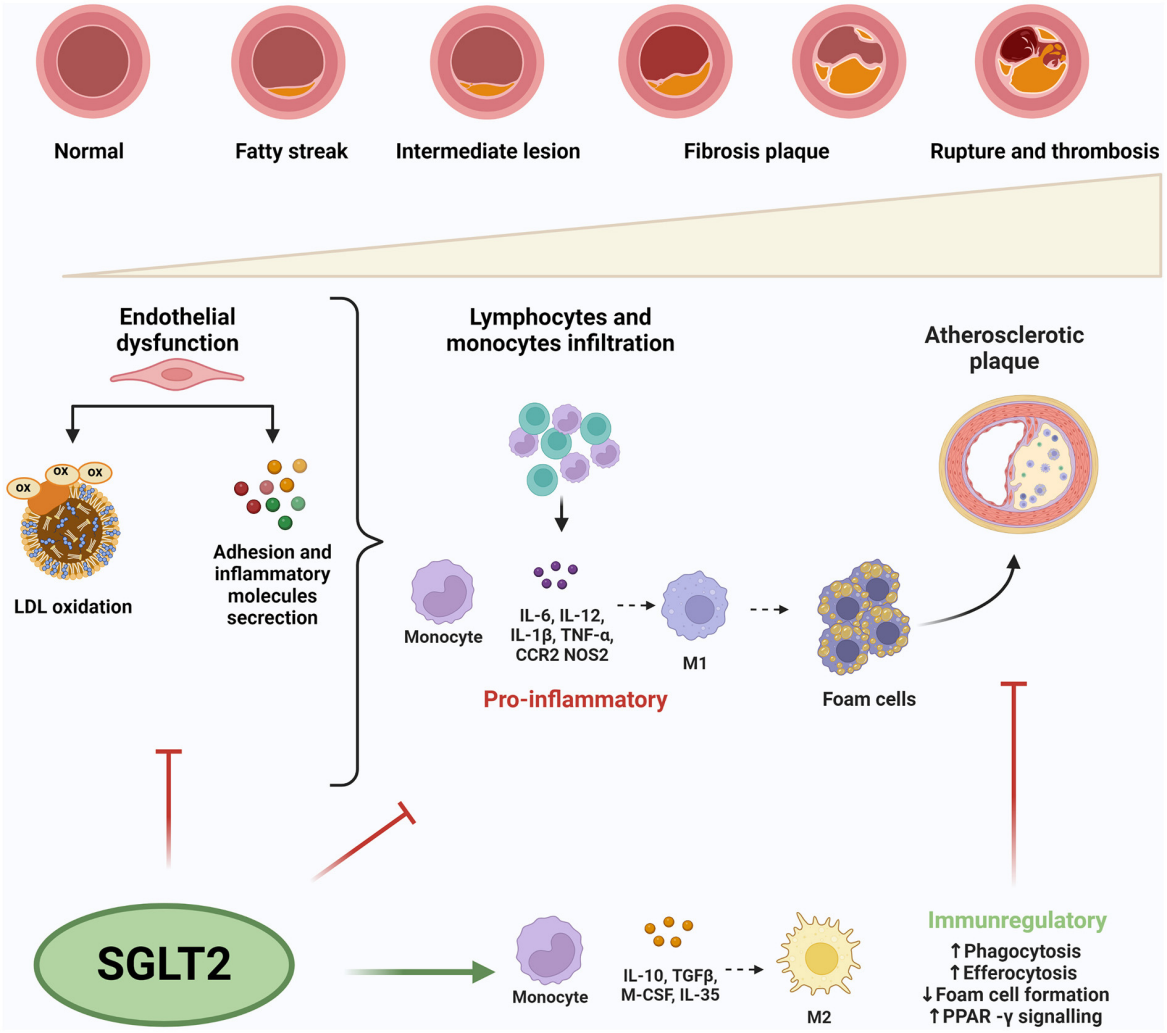
Sodium-glucose co-transport 2 inhibitors' effects on inflammation in atherosclerosis. SGLT2i inhibit endothelial dysfunction reducing the expression of circulating inflammatory molecules and LDL-oxidation. Moreover, SGLT2i attenuate macrophages infiltration, M1 polarization and foam cell formation. M1 macrophages express the main pro-inflammatory molecules playing role in maintaining chronic inflammation, forming foam cells, and plaque initiation and progression Inversely, M2 macrophages are associated with an anti-inflammatory phenotype producing anti-inflammatory factors. The imbalance of these,polarized macrophages may be responsible for plaque development or regression. ox-LDL, oxidized-LDL; ILs, interleukins; TNF-α, tumor necrosis factor-α; CCR2, C-C chemokine receptor type 2; NOS 2, nitric oxidase synthase 2; TGF-β, transforming growth factor beta; M-CSF, macrophage colony-stimulating factor.

Inversely, M2 macrophages are associated with an anti-inflammatory phenotype being polarized in response to IL-4 and IL-13 and producing anti-inflammatory factors such as the IL-1 receptor agonist, transforming growth factor beta (TGF-β), and IL-10 (13, 15). Consequently, the imbalance of these polarized macrophages may be responsible for plaque development or regression (16) (Figure 1).

In addition, the lipids accumulation in macrophages, with associated foam cell formation, results in the activation of the nucleotide-binding domain-like receptor protein 3 (NLRP3) inflammasome complex, which promotes the release of pro-inflammatory cytokines contributing to the development and progression of atherosclerosis (17). Indeed, inflammasome activation causes the amplification of the inflammatory response by promoting the expression of adhesion molecules, a proliferation of vascular smooth muscle cells, and the activation of macrophages (18).

Moreover, in the atherogenesis process, oxidative stress and cellular senescence contribute to the maintenance of inflammation and endothelial dysfunction. Indeed, reactive oxygen species (ROS) induce both the synthesis of pro-inflammatory cytokines and stimulate the expression of adhesion molecules, thus allowing monocytes to transmigrate into the vessel wall (19). In addition, ROS can promote the expression of scavenger receptors on vascular smooth muscle cells, promoting lipid accumulation and transformation into foamy cells (17).

Senescence-inducing stress can result from various cardiovascular risk factors (20). Indeed, senescent cells exhibit a specific senescence-associated secretory phenotype (SASP), which consists of inflammatory cytokines, chemokines, growth factors, and proteases (21). The accumulation of senescent cells progressively results in chronic low-grade inflammation, termed “inflammaging” (21). Senescent endothelial cells show altered permeability and nitric oxide (NO) production at the vascular level, inducing endothelial dysfunction. Senescent vascular smooth muscle cells, on the other hand, show reduced proliferation and an increased tendency to apoptosis. Therefore, the accumulation of senescent cells in atherosclerotic lesions may promote plaque progression (22).

In the second stage, characterized by plaque progression, smooth muscle cells produce extracellular matrix components contributing to plaque thickening and progressive growth in the vessel's lumen. In addition, macrophages and smooth muscle cells undergo apoptosis by going on to form a central lipid-rich core. Therefore, in the late stage, the plaque consists of a central lipid core and a fibrous cap (23). Pro-inflammatory cytokines, such as IL-1, IL-6, Interferons (IFN) gamma, metalloproteases-2 and 9 (MMP-2 and MMP-9), and metallopeptidase inhibitors (TIMP-1, TIMP-2), are responsible for plaque erosion/rupture. Specifically, IL-6 plays a crucial role in inducing a prothrombotic state by positive regulation of plasminogen activator inhibitor type 1 (PAI-1) during the acute inflammation phase response and negative regulation of antithrombin and protein S (24). Moreover, IL-6 induces the up-regulation of adhesion molecules in the endothelial cells and increases vascular permeability and cellular dysfunction (25).

Therefore, it is possible to distinguish two types of plaques:

- Stable plaques, which possess a thick fibrous cap. It can regress with the proper lifestyle and/or drug therapy or may progress by occluding the vessel lumen.
- Vulnerable or unstable plaques are rich in macrophages and possess a thick lipid core and a thin fibrous cap. It is more likely to undergo rupture and complications (26).

As a result, the cardiac complications of atherosclerotic disease are related to (I) reduced blood flow due to an insufficient oxygen supply, such as angina pectoris; (II) plaque can occlude the vessel leading to ischemia of downstream tissues, for example, in myocardial infarction or stroke; (III) detachment of part of the thrombus, termed an embolus can result in occlusions of distal arteries as pulmonary embolism (7).

## SGLT2 inhibitors

The SGLT2i are a new category of anti-diabetic drugs, approved by the Food and Drug Administration (FDA), used to treat patients with type 2 diabetes (27). SGLT2i family includes dapagliflozin, empagliflozin, luseogliflozin, ipragliflozin, phlorizin, and canagliflozin (28).

Sodium-glucose co-transport 2 inhibitors, targeting the major glucose transporter SGLT2 in the kidney, block glucose reabsorption in the proximal renal tubule, increasing glycosuria independently of insulin sensitivity and secretion (29). The SGLT2 inhibition reduces glucose reabsorption, promotes urinary glucose secretion, and causes a negative caloric balance (30).

Phlorizin was the first natural SGLT2i isolated from the root bark of apple trees. Phlorizin, binding the extracellular surface of SGLT1 and SGLT2 in the presence of Na^+^, inhibits proteins in a reversible, competitive way (31). However, caused of its poor solubility in water and low absorption in the gastrointestinal tract, several molecules with a similar structure were subsequently developed (32).

Many compounds with increased stability, bioavailability, and high selectivity for SGLT2 over SGLT1 have been identified. Currently, only four molecules are licensed by the European Medicines Agency (EMA) and the FDA: canagliflozin, dapagliflozin, and empagliflozin (33).

In addition to structural differences, these compounds present variable selectivity to SGLT1 and SGLT2. Empagliflozin has the greatest selectivity for SGLT2 (>2,500-fold) compared to SGLT1. The binding affinity of dapagliflozin for SGLT2 is 100-fold higher than that for SGLT1, and Canagliflozin shows an SGLT2 selectivity of 250-fold (34).

Despite different selectivity, SGLT2i share similar pharmaco-kinetic characteristics, such as rapid oral absorption, a long half-life, extensive hepatic metabolism, low renal elimination, and the absence of drug-drug interactions (28).

Besides their effects on blood glucose, preclinical and clinical studies have demonstrated that SGLT2i therapy provides extra-glycemic effects and clinical advantages in patients with diabetes (35), such as improved metabolic control and cardiac and renal protection. In particular, being renal proximal, the mainly SGLT2i target, these drugs alleviate albuminuria and reduce the glomerular filtration rate (eGFR) and the plasma urate levels (36).

In addition, SGLT2 inhibitors have been convincingly demonstrated to reduce main adverse cardiovascular events and the occurrence of heart failure with reduced ejection fraction (HFrEF), independently of glycemic status (37). Interestingly, these cardio-protective effects appear independent of glucose-control efficacy and attributable to their direct and indirect action on organs (38). In particular, cardiovascular protection is related to hemodynamic mechanisms and the metabolic and anti-inflammatory effects. Indeed, SGLT2i improves lipid profile, reduces blood pressure, uricemia, visceral fat, body weight, and inflammatory markers, and reduces cardiovascular events and mortality (39–41).

Several studies have been conducted to establish underlying mechanisms of SGLT2i cardio-protective effects. In particular, studies conducted in animal models and patients with diabetes demonstrated the anti-atherosclerotic action of SGLT2i, revealing some potential mechanisms.

Nevertheless, the molecular mechanisms are still unclear (42). This gap is mainly due to the absence of evidence showing SGLT2 expression in monocytes/macrophages and endothelial cells.

The mechanisms underlying the SGLT2 benefit on atherosclerosis could be multifactorial and due to attenuation of secretion of inflammatory molecules, reduction of macrophage infiltration, improvement of autophagy impairment, and inhibition of endothelial dysfunction (43) out of their glucose-lowering effects (44).

Therefore, considering the clinically beneficial effects of SGLT2 on atherosclerosis, we provide an insightful overview focusing on mechanisms by which SGLT2i may act on inflammation in atherosclerosis and atherosclerosis cardiovascular diseases.

## Effects of SGLT2 inhibitors on inflammation in atherosclerosis

As described before, inflammation is a significant factor in vascular cell dysfunction, causing the development and progression of atherosclerosis in diabetes (45).

The atherosclerosis treatment from an inflammatory perspective could be a valid therapeutic strategy. Clinical trials have demonstrated that modulation of inflammation can prevent atherosclerosis and its complications.

Recent studies have shown an improvement in inflammatory and oxidative status in subjects with T2DM treated with SGLT2i regardless of glycemic control (46). These data support the hypothesis that SGLT2i show cardio-protective effects action on inflammation.

Therefore, inflammation may be considered a mechanism by which SGLT2i can exert their protective effects against atherosclerosis. This role can be discussed regarding the impact of SGLT-2i on systemic inflammation and immune signaling pathways, including changes in local atherosclerotic tissues.

### Experimental evidence

Experimental evidence revealed that SGLT2i reduced the expression of circulating inflammatory molecules, such as TNF-α, monocyte chemoattractant protein 1 (MCP-1), platelet endothelial cell adhesion molecule-1 (PECAM-1), VCAM-1, intercellular adhesion molecule 1 (ICAM-1), IL-1β, and IL-6 (47–50). Several studies have described potential mechanisms by which SGLT2i inhibit the expression of inflammatory molecules. In cultured human endothelial cells, canagliflozin impeded the release of IL-6 and MCP-1 induced by IL-1β in an 5' adenosine monophosphate-activated protein kinase (AMPK)-dependent manner (51).

Human umbilical vein endothelial cells (HUVECs) and macrophages exposed to dapagliflozin and LPS showed attenuate levels of LPS-induced TLR-4 expression, NF-κB p65 phosphorylation, and miR-155 and elevated levels of miR-146a. Moreover, dapagliflozin shifted from inflammatory M1 macrophages toward M2-dominant macrophages (52).

In LPS-stimulated RAW 264.7 macrophages, empagliflozin in association with gemigliptin and Dipeptidyl peptidase-4 (DPP-IV) inhibitor reduced gene expression and pro-inflammatory cytokine and chemokine release through the IKK/NF-κB/JAK2 - STAT1/3, and MKK4/7- JNK pathways (53).

Similar results were also obtained in mouse models. In nicotinamide and streptozotocin (NA/STZ)-treated ApoE KO mice, the treatment with luseogliflozin reduces the expression of inflammation-related genes, including F4/80, TNFα, IL-1β, IL-6, ICAM-1, PECAM-1, MMP2 and MMP9 (47).

In STZ-treated ApoE–/– mice, dapagliflozin inhibited IL-1β and IL-18 secretion, blocking the ROS-NLRP3-caspase-1 pathway showing that NLRP3 inflammasome complex could be considered an SGLT2i target (49, 54).

Two mechanisms by which empagliflozin inhibited NLRP3 activation were identified, one is dependent on β-hydroxybutyrate (BHB), and the other is Ca+ dependent. Specifically, *ex vivo* experiments with macrophages demonstrated that empagliflozin inhibited NLRP3, increasing BHB levels and reducing glucose, uric acid, and insulin (55). Empagliflozin reduced Ca^+^ levels by attenuating Na^+^ intracellular accumulation, causing the NLRP3 inhibition and improving functional recovery (56).

Moreover, it was demonstrated that also dapagliflozin reduced the production of NLRP3 protein by activating mTOR Complex 2 (mTORC2), leading to the activation of AMPK and Forkhead box 3 (FOXO3) (57, 58).

Considerable evidence showed that SGLT2i ameliorated endothelial dysfunction and improved endothelium-dependent vasodilation. In particular, in diabetic ApoE –/– mice, empagliflozin improved endothelial function by reducing CD68, MCP-1, ICAM-1, and TNF-α, suppressing the development and progression of atherosclerotic lesions (59). In a High-fat diet (HFD)-induced obese C577BL/6J MICE, empagliflozin increased plasma levels of Fibroblast Growth Factor 21 (FGF21), protecting the cells from damage caused by atherosclerosis-associated oxidative stress (60). Ipragliflozin reduced oxidative stress markers, thiobarbituric acid reactive substances (TBARS), and inflammation proteins (CRP, TNF-α, IL-6, and MCP-1) in streptozotocin-nicotinamide-induced diabetic mice (61). In abdominal aortic aneurysm induced by Angiotensin II infusion in apolipoprotein mice, the treatment with empagliflozin inhibited leukocyte-endothelial cell interactions, macrophages infiltration, and secretion of pro-inflammatory markers such as chemokine (C-C motif) ligand 2 (CCL-2), CCL-5, Vascular Endothelial Growth Factor (VEGF), MMP-2, MMP-9, p38 mitogen-activated protein kinase (MAPK), and NF-kB (62).

Evidence revealed that SGLT2i attenuates macrophage infiltration and inflammation, foam cell formation, and M1 polarization, crucial steps in the development of atherosclerosis. It was demonstrated that empagliflozin reduced macrophage infiltration and CD36 gene expression resulting in the loss of macrophage capability to scavenger ox-LDL and foam cell formation in db/db mice (63). A similar result was obtained in the streptozotocin (STZ)-induced diabetic model, where the macrophages proliferation and leukocyte adhesion were significantly decreased in empagliflozin and dapagliflozin -treated mice with a subsequent reduction in the plaque size (51, 64).

Moreover, dapagliflozin reduced macrophage infiltration and induced M2 polarization with a concomitant decrease in M1 through a mechanism depending on a RONS-dependent STAT3 pathway. These data demonstrated that dapagliflozin induced the production of anti-inflammatory factors participating in inflammation prevention and tissue repair (65).

Similar results were demonstrated in white rabbits, where dapagliflozin increased M2 macrophages and inhibited the expression of TLR4 and NF-κB (66).

However, the exact mechanism by which SGLT2i can induce M2 polarization and reduce macrophage infiltration is not fully elucidated, and many possible hypotheses are formulated. Indeed, reducing glucose levels, the principal energy source of macrophages, might have a role (35) (Figure 1; Table 1).

**Table 1:** Experimental evidence of atheroprotective effects in animal models.

**Drugs**	**Experimental model**	**Treatment**	**Possible atheroprotective effects**	**References**
Canaglifozin	HUVECs	10 μmol/L Canaglifozin for 30 min	Inhibition IL-1β-stimulated adhesion of pro-monocytic U937 cells and secretion of IL-6 and monocyte chemoattractant protein-1 (MCP-1)	(51)
Dapaglifozin	HUVECs	Dapaglifozin (1 mg/kg/day) and lipopolysaccharide (LPS 20 ng/ml) for 24 h under normal (5.5 mmol/L, NG) or high glucose (25 mmol/L, HG) conditions	↓ LPS-induced TLR-4 expression, NF-κB p65 phosphorylation, miR-155 and miR-146a Shift from M1 macrophages to M2-dominant macrophages	(52)
Empaglifozin	RAW 264.7 murine	40, 60, and 80 μM for 4 h	↓ pro-inflammatory cytokine and chemokine	(53)
Luseogliflozin	NA/STZ-treated ApoE KO mice	Dose with maximal glucose-lowering efficacy for 1 week	↓ TNFα, IL-1β, IL-6, ICAM-1, PECAM-1, MMP2, and MMP9	(47)
Dapaglifozin	STZ-treated ApoE–/– mice	1.0 mg/kg/day for 12-week	Inhibition ROS-NLRP3-caspase-1 pathway	(49, 54)
Dapaglifozin	Type 2 diabetic (BTBR ob/ob) and wild-type (WT) mice	Dapaglifozin, or Dapaglifozin (1 mg/kg/day) + Saxagliptin (10 mg/kg/day) for 8 weeks	↓ Activation of the Nlrp3/ASC inflammasome	(57, 58)
Empaglifozin	Diabetic ApoE –/– mice	(20 mg/kg/day) for 8 or 12 weeks	↓ CD68, MCP-1, ICAM-1, and TNF-α → suppressing the development and progression of atherosclerotic lesions	(60)
Ipragliflozin	NA/STZ-induced diabetic mice	10 mg/kg/day for 10 weeks	↓ ROS, TNF α, IL-6, CRP, MCP-1	(61)
Empaglifozin	(HFD)-induced obese C577BL/6J mice	HFD + Lo Empa, equivalent to 3 mg/kg bodyweight HFD + Hi Empa, equivalent to 10 mg/kg bodyweight	↑ FGF21	(60)
Empaglifozin	Apolipoprotein mice	3 mg/kg per day	↓ CCL-2, CCL-5, VEGF, MMP-2, MMP-9, p38 MAPK, and NF-kB	(62)
Dapaglifozin	Non-diabetic male Wistar rats (200–250 g)	Dapagliflozin (0.1 mg/kg per day), phlorizin (0.4 g/kg per day), dapagliflozin + S3I-201 (a STAT3 inhibitor), or phlorizin + S3I-201 for 4 weeks	↓ M1 by RONS-dependent STAT3-pathway	(65)
Dapaglifozin	Rabbit	1 mg/kg/day for 8 weeks	↓ Expression of TLR4 and NF-κB ↑ M2 macrophages	(66)

CRP, C reactive protein; HDF, a high-fat diet; ICAM-1, intercellular cell adhesion molecule-1; LDLR, low-density lipoprotein receptor; IL-1β, interleukin-1β; IL-6, interleukin-6; MCP-1, monocyte chemoattractant protein-1; MMP, matrix metalloproteinase; NF-κB, nuclear factor-κB; NLRP3, nucleotide-binding domain-like receptor protein 3ROS, reactive oxygen species; TNF-α, tumor necrosis factor-α; VEGF, Vascular endothelial growth factor; VCAM-1, vascular cell adhesion molecule-1.

### Clinical evidence

Several clinical studies have been conducted to evaluate the changes in the main inflammatory and oxidative stress biomarkers to evaluate the potential role of SGLT2i in protecting against atherosclerosis. The inflammatory markers implicated and therefore assessed in the atherosclerosis process are CRP, IL-6, and TNF-α.

In a single-center, open-label, randomized, prospective study, the administration of empagliflozin (10 mg/day for 12 months) to 51 diabetic patients induced a significant reduction of blood hs-CRP levels associated compared with baseline and placebo (−74.4% vs. placebo and −55.6% vs. baseline) (67).

Similarly, the CANOSSA trial, a prospective and open-label study that enrolled 35 patients with diabetes mellitus and stable chronic heart, demonstrated that the canagliflozin administration (100 mg/day for 12 months) induced a significant decrease in hs-CRP after 3, 6, and 12 months compared with baseline (3 months: *p* = 0.002, 6 months: *p* = 0.001, 12 months: *p* = 0.007) (68).

A comparative efficacy study between canagliflozin and empagliflozin was also conducted on 32 diabetic patients to evaluate the effect on inflammatory cytokines. The results suggest that treatment with empagliflozin 10 mg/day for 6 months is more effective in reducing inflammatory cytokines IL-6 (*p* = 0.002 vs. *p* = 0.27) and TNF-alpha (*p* = 0.002 vs. *p* = 0.29), while canagliflozin was more effective in reducing HbA1c (69).

DEFENSE STUDY, a prospective, randomized, open-label, blinded-endpoint, parallel-group, evaluated dapagliflozin's effectiveness on vascular endothelial function and glycemic control in subjects affected by T2DM early-stage. Dapagliflozin, in association with metformin for 16 weeks, improved endothelial function and significantly decreased urine 8-OHdG/creatinine, a marker of oxidative stress, compared to the only metformin group (*p* < 0.001) (70).

Moreover, atherosclerotic plaques of diabetic patients treated with canagliflozin displayed increased SIRT 6 expression and lower oxidative stress and inflammation markers (71) (Table 2).

**Table 2:** Clinical evidence of SGLT2i's atheroprotective effects.

**Drugs**	**Type of study**	**Characteristics of patients (number)**	**Treatment**	**Possible atheroprotective effects**	**References**
Empaglifozin	Prospective, open-label observational	T2DM (15)	24-week empagliflozin 10 mg vs. baseline	↓ CRP/hs-CRP	(52)
Canaglifozin	Prospective, open-label, randomized controlled trial	T2DM, HF (35)	12 month CRP canagliflozin 100 mg vs. baseline	↓ CRP/hs-CRP	(53)
Dapaglifozin	Prospective, open-label, blinded endpoint, randomized	T2DM (72)	16 week dapagliflozin 5 mg/day + Metformin 750 mg/day vs. Metformin 1,500 mg/day	↓ 8-OHdG	(55)

CRP, c-reactive protein; TNF α, tumor factor alpha necrosis; IL6, interleukin-6; hsCRP, high-sensitivity c-reactive protein; 8-OhdG, 8-Hydroxy-20–deoxyguanosine; T2DM, type 2 diabetes mellitus.

## Effects of SGLT2i on atherosclerosis cardiovascular diseases and cardiovascular mortality

Atherosclerotic cardiovascular diseases (ASCVD) represent a very important cause of death and disability, especially in subjects affected by T2DM. The main manifestations of ASCVD are coronary heart disease, ischemic stroke, peripheral artery disease, and heart failure (73).

### Coronary artery disease

Coronary artery disease (CAD) is the most common atherosclerotic vascular disease, and it includes two main clinical phenotypes: stable/unstable angina and acute MI (AMI). Data regarding MI in patients treated with SGLT2i are conflicting. In the EMPA-REG OUTCOME study and CANVAS study, no significant difference in MI incidence was observed between the treated and placebo group (RR 0.87; 95% CI 0.70–1.09 and RR 0.85; 95% CI 0.69–1.05, respectively) (74, 75). However, the sub-analysis of the CVD-REAL study and the CVD-REAL2 study showed a lower risk of MI associated with SGLT2i therapy (RR 0.85; 95% CI 0.72–1.00) (RR 0.81; 95% CI 0.74–0.88) (76, 77). A meta-analysis, including 40 trials and ~60,000 participants, found a 14% reduction in the incidence of MI in SGLT2i users compared to controls (78). Nevertheless, no reduction was found in the occurrence of angina pectoris (79).

The effects of early SGLT2i treatment in patients with recent AMI were not well-studied.

Early SGLT2i treatment might improve cardiovascular outcomes through its beneficial effects on endothelial function, neurohormonal activation, cardiomyocyte necrosis, and reperfusion injury (80). Three studies are evaluating the efficacy and safety of SGLT2i in patients with AMI (80):

- EMMY (Impact of EMpagliflozin on Cardiac Function and Biomarkers of Heart Failure in Patients With Acute Myocardial Infarction) trial (NCT03087773)
- DAPA-MI (Dapagliflozin Effects on Cardiovascular Events in Patients With an Acute Heart Attack) (NCT04564742)
- EMPACT-MI (A Streamlined, Multicenter, Randomized, Parallel Group, Double-blind Placebo-controlled Superiority Trial to Evaluate the Effect of EMPAgliflozin on Hospitalization for Heart Failure and Mortality in Patients With Acute Myocardial Infarction) trial (NCT04509674).

### Heart failure

The recent European clinical guidelines on HF have approved SGLT2 inhibitors for treating HFrEF and preventing HF hospitalizations independently of diabetes (37, 72, 81–83). In particular, EMPA-REG OUTCOME and the CANVAS study showed a significant reduction in hospitalizations for HF in patients treated with empagliflozin and canagliflozin, respectively, vs. placebo (RR 0.65; 95% CI 0.50–0.85) (RR 0.67; 95% CI 0.52–0.87) (75, 84). DECLARE-TIMI 58 showed a reduced hospitalization rate for HF compared to the placebo group (RR 0.73; 95% CI 0.61–0.88) (85).

However, in the VERTIS-CV study, the reduction of HF hospitalization was not statistically significant because the endpoint was a composite of cardiovascular death and hospitalization for HF (86).

Other studies, such as DAPA-HF and EMPEROR-Reduced, also provide convincing evidence of the beneficial effects of SGLT2i on HF (72, 87). A meta-analysis of these studies confirmed the important role of empagliflozin and dapagliflozin for reducing all-cause and cardiovascular mortality and HF hospitalizations and slowing the progression of renal disease (88). A meta-analysis, including 17 studies with ~51,348 participants, suggests a 32% reduction of HF risk (RR, 0.68; 95%CI, 0.63–0.73) in SGLT2-i users (78).

### Stroke

Data regarding the risk of stroke in patients treated with SGLT2i are conflicting. The EMPAREG OUTCOME study did not show a significant reduction in the incidence of stroke in patients treated with empagliflozin compared to the placebo group (RR 1.18; 95% CI 0.89–1.56) (89). The same result was found in CANVAS and DECLARE-TIMI 58 studies (RR 0.9; 95% CI 0.71–1.15 and RR 1.01; 95% CI 0.84–1.21, respectively) (75, 85).

However, the CVD-REAL and the CVD-REAL2 studies showed a lower risk of stroke associated with SGLT2i therapy (RR 0.83; 95% CI 0.71–0.97) (RR 0.68; 95% CI 0.55–0.84) (76, 77).

However, the analysis conducted by these studies did not consider the SGLT2i effect on different types of stroke (fatal, non-fatal, ischemic, hemorrhagic, and TIA), making the data conflicting.

Therefore, a systematic review and meta-analysis were performed to evaluate the stroke subtypes. Considering five studies, CANAVAS, CREDENCE, DECLARE-TIMI, EMPAREG AND VERTIS- CV, they found that SGLT2i reduced the incidence of hemorrhagic stroke and Atrial Fibrillation (AF) by 0.5%. This could be related to the natriuretic effect and subsequent reduction in blood pressure. However, Further studies aimed at evaluating the impact of SGLT2 on this pathology will be needed (90).

### Peripheral artery disease

Peripheral artery disease (PAD) is a manifestation of systemic atherosclerosis, characterized by progressive narrowing to occlusion of arterial vessels in the limbs, resulting in ulceration and subsequent gangrene and amputation.

Peripheral artery disease increases the risk of coronary artery and cerebrovascular disease and is an independent predictor of CVD death (91). The EMPAREG study, although it did not consider PAD as a primary and secondary endpoint, revealed that Empaglifozin resulted in significant benefits in subjects with PAD, which no reported amputations.

However, the CANAVAS and CANAVAS-R studies reported that amputation risk was twice in the treated group compared to the placebo group (6.3 cases per 1,000 patients per year) (75). Although the molecular mechanisms responsible for this have not yet been elucidated, the EMA has reported the potential increased risk of lower limb amputation (affecting mainly the toes) in patients taking SGLT2 inhibitors.

### Major adverse cardiac events

The most important cardiovascular trials for SGLT2 evaluated the incidence of major adverse cardiac events (MACEs), such as cardiovascular death, non-fatal myocardial infarction, and non-fatal stroke. A meta-analysis of 6 placebo-controlled clinical outcomes trials (EMPA-REG OUTCOME, CANVAS, DECLARE-TIMI 58, CREDENCE, VERTIS CV) (85, 86, 92, 93), including a total of 46 969 patients (66.2% with prevalent ASCVD) suggests that SGLT2 inhibitors significantly reduced the risk of MACE (HR, 0.90; 95% CI, 0.85–0.95;); the presence or absence of ASCVD did not modify the treatment outcome on MACE (HR, 0.89; 95% CI, 0.83–1.07) (94).

### Cardiovascular mortality

Four large cardiovascular outcome studies have recently been completed: EMPAREG OUTCOME, CANVAS, DECLARE-TIMI, and VERTIS CV (85, 86, 92, 93). These studies showed a reduction in overall and cardiovascular mortality (CV Mortality), which was significant in the EMPA-REG and CANVAS studies but not in DECLARE-TIMI 58. Specifically, the CV mortality rate was lower in the EMPA-REG OUTCOME study. in patients treated with SGLT2i compared to placebo (RR 0.62; 95% CI 0.49–0.77) (89). CV mortality rate was not significantly reduced in CANVAS, DECLARETIMI 58 and VERTIS-CV (RR 0.87; 95% CI 0.72–1.06 and RR 0.98; 95% CI 0.82–1.17, HR 0.89 95% CI 0.73–1.05, respectively) (75, 95). A recent meta-analysis showed no significant effect on CV mortality associated with SGLT2i treatment (except Empagliflozin) compared to placebo (OR 0.87; 95% CI 0.63–1.21) (96) (Table 3).

**Table 3:** Cardiovascular outcomes of SGLT2 inhibitors trials.

	**EMPA-REG**	**CANVAS**	**CREDENCE**	**DECLARE-TIMI 58**	**DAPA–HF**	**VERTIS-CV**
References	(74)	(60)	(77)	(69)	(66)	(78)
Drugs	Empaglifozin	Canagliflozin	Canagliflozin	Dapagliflozin	Dapagliflozin	Ertugliflozin
Study population	T2DM patients with CVD	T2DM patients with CVD or CV risk factors	T2DM patients with CKD	T2DM patients with ASCVD or CV risk factors	Patients with rHF	T2DM patients with ASCVD
Number of patients	7,020	10,142	4,401	17,150	4,744	8,246
Median follow-up	3.1 years	2.4 years	2.6 years	4.2 years	18.2 months	3.5 years
**CV outcomes**
MACE	0.86 (0.74–0.99)	0.86 (0.75–0.97)	0.80 (0.67–0.95)	0.93 (0.84–1.03)	–	0.97 (0.85–1.11)
CV death	0.62 (0.49–0.77)	0.87 (0.72–1.06)	–	0.98 (0.82–1.17)	0.82 (0.69–0.98)	0.92 (0.77–1.11)
CV death or HHF	0.66 (0.55–0.79)	–	–	0.83 (0.73–0.95)	0.75 (0.65–0.85)	–

ASCVD, atherosclerotic cardiovascular diseases; CKD, chronic kidney disease; CV, cardiovascular; CVD, cardiovascular diseases; HF, heart failure; HHF, hospitalization for heart failure; MACE, major adverse cardiovascular events: including death from cardiovascular causes, non-fatal MI, and non-fatal stroke; T2DM, type 2 diabetes mellitus.

## From clinical impact to future prospective

Sodium-glucose co-transport 2 inhibitors exert anti-atherosclerotic properties attenuating inflammatory factors, alleviating inflammation, mitigating insulin resistance, and reducing stress on vessels, inhibiting atherosclerosis development and progression (46). Recent clinical trials have analyzed the landmark cardiovascular outcomes, showing that SGLT2i may reduce MI, heart failure and HF hospitalization, MACE, and cardiovascular death in subjects affected by T2DM (81, 86–89). Nevertheless, data regarding MI, stroke, and PAD are conflicting, probably because the analyzed population is heterogeneous, the different stroke subtypes were not considered, different molecules were analyzed, and the follow-up time is short. Therefore, observational studies of high quality, with an adequate number of events and follow-up times, should be conducted to examine the potential role of SGLT2i in subclinical atherosclerosis and ASCVD events in subjects affected and not by T2DM. Despite conflicting data, SGLT2i represents a promising drug class; important innovation is reported in European guidelines on diagnosing and treating heart failure. SGLT2i is used as a class I recommendation in treating HFrEF (72).

Moreover, cardiovascular safety studies conducted with SGLT2i suggested that subjects affected by T2DM could benefit from SGLT2i treatments with positive effects on cardiovascular events (97). Unfortunately, only a small number of subjects affected by T2DM are treated with SGLT2i, whereas their widespread use would reduce deaths and hospitalizations each year. We think using these drugs will lead to a global evaluation of patients regarding glycemic control and cardiac function. Moreover, its use will have a substantial cost reduction and hospitalization advantage.

## Conclusions

Atherosclerosis is a chronic inflammatory disorder representing the major potential risk factor of CVDs. The progress in managing the atherosclerosis complications has extended life, but many individuals still present impaired cardiac function, straining healthcare systems and resources (7). In this review, we emphasize SGLT2i by providing updated information about its implication in atherosclerosis and ASCVD. SGLT2i exerts anti-atherosclerotic properties attenuating inflammatory factors and reducing MI, heart failure MACE in subjects affected by T2DM.

Therefore, in conclusion, SGLT2 inhibitors can represent up-and-coming therapeutic drugs with pleiotropic effects in terms of metabolic control and reduction of associated cardiovascular complications.

## Author contributions

LSc, VC, and MB: concept and design. LSc, VC, FT, RF, APe, PG, MF, APu, AD, LSp, RM, and MB: drafting of the manuscript. All authors have read and agreed to the published version of the manuscript.

## Funding

This study was funded by PON Ricerca e Innovazione 2014–2020 ARS01_01270.

## Conflict of interest

The authors declare that the research was conducted in the absence of any commercial or financial relationships that could be construed as a potential conflict of interest.

## Publisher's note

All claims expressed in this article are solely those of the authors and do not necessarily represent those of their affiliated organizations, or those of the publisher, the editors and the reviewers. Any product that may be evaluated in this article, or claim that may be made by its manufacturer, is not guaranteed or endorsed by the publisher.

## Abbreviations

8-OHdG: 8-hydroxy-2'-deoxyguanosine
AMI: acute MI
AMPK: 5’ adenosine monophosphate-activated protein kinase
ASCVD: atherosclerotic cardiovascular diseases
BHB: β-hydroxybutyrate
CAD: coronary artery desease
CCL: chemokine (C-C motif) ligand 2
CCR2: C-C chemokine receptor type 2
CD68: cluster of differentiation 68
CRP: C-reactive protein
CVD: cardiovascular disease
CXCL: chemokines ligand
DAMPs: damage-associated molecular patterns
DPP-IV: dipeptidyl peptidase-4
eGFR: glomerular filtration rate
EMA: European Medicines Agency
FDA: Food and Drug Administration
FGF21: fibroblast growth factor 21
FOXO 3: forkhead box
HF: heart failure
HFD: high-fat diet
HFrEF: heart failure with reduced ejection fraction
ICAM-1: intercellular adhesion molecule 1
IFN: interferons
IKK: IkappaB kinase
IL-1R1: interleukin-1 Receptor 1
ILs: interleukins
JAK2: Janus kinase
JNK: c-Jun N-terminal kinases
LDL: low density lipoprotein
LPS: lipopolysaccharide
MACE: major adverse cardiac event
MAPK: mitogen-activated protein kinase
MCP-1: monocyte chemoattractant protein 1
MI: myocardial infarction
MKK: protein kinase kinase
MMPs: metalloproteases
mTORC2: mTOR complex 2
NA/STZ: nicotinamide and streptozotocin
NF-kB: nuclear factor kappa-light-chain-enhancer of activated B cells
NLRP3: nucleotide binding domain-like receptor protein 3
NO: nitric oxide
NOS 2: nitric oxidase synthase 2
ox-LDL: oxidized-LDL
PAD: peripheral artery disease
PAI-1: plasminogen activator inhibitor type 1
PECAM-1: platelet endothelial cell adhesion molecule-1
ROS: reactive oxygen species
SASP: senescence-associated secretory phenotype
SGLT2i: sodium-glucose co-transport 2 inhibitors
SIRT: sirtuin
STAT: signal transducer and activator of transcription
STZ: streptozotocin
T2DM: type 2 diabetes mellitus
TBARS: thiobarbituric acid reactive substances
TGF-ß: transforming growth factor beta
TIMPs: metallopeptidase inhibitor 1
TLR: toll-like receptors
TNF-α: tumor necrosis factor- α
VCAM-1: vascular cell adhesion molecule 1
VEGF: vascular endothelial growth factor.
